# The multi-target aspect of an MmpL3 inhibitor: The BM212 series of compounds bind EthR2, a transcriptional regulator of ethionamide activation

**DOI:** 10.1016/j.tcsw.2021.100068

**Published:** 2021-11-23

**Authors:** Alice R. Moorey, Alejandro Cabanillas, Sarah M. Batt, Sonja Ghidelli-Disse, Beatriz Urones, Olalla Sanz, Joel Lelievre, Marcus Bantscheff, Liam R. Cox, Gurdyal S. Besra

**Affiliations:** a Institute of Microbiology and Infection, School of Biosciences, University of Birmingham, Birmingham B15 2TT, U.K; b Diseases of the Developing World, GlaxoSmithKline, Severo Ochoa 2, Tres Cantos, Madrid 28760, Spain; c Cellzome – a GSK Company, Meyerhofstrasse 1, 69117 Heidelberg, Germany; d School of Chemistry, University of Birmingham, Edgbaston, Birmingham B15 2TT, U.K

**Keywords:** *Mycobacterium tuberculosis*, MmpL3, EthR2, EthA2, BM212

## Abstract

The emergence of drug-resistant strains of *Mycobacterium tuberculosis* (*Mtb*) ensures that drug discovery efforts remain at the forefront of TB research. There are multiple different experimental approaches that can be employed in the discovery of anti-TB agents. Notably, inhibitors of MmpL3 are numerous and structurally diverse in *Mtb* and have been discovered through the generation of spontaneous resistant mutants and subsequent whole genome sequencing studies. However, this approach is not always reliable and can lead to incorrect target assignment and requires orthogonal confirmatory approaches. In fact, many of these inhibitors have also been shown to act as multi-target agents, with secondary targets in *Mtb*, as well as in other non-MmpL3-containing pathogens. Herein, we have investigated further the cellular targets of the MmpL3-inhibitor BM212 and a number of BM212 analogues*.* To determine the alternative targets of BM212, which may have been masked by MmpL3 mutations, we have applied a combination of chemo-proteomic profiling using bead-immobilised BM212 derivatives and protein extracts, along with whole-cell and biochemical assays. The study identified EthR2 (Rv0078) as a protein that binds BM212 analogues. We further demonstrated binding of BM212 to EthR2 through an *in vitro* tryptophan fluorescence assay, which showed significant quenching of tryptophan fluorescence upon addition of BM212. Our studies have demonstrated the value of revisiting drugs with ambiguous targets, such as MmpL3, in an attempt to find alternative targets and the study of off-target effects to understand more precisely target engagement of new hits emerging from drug screening campaigns.

## Introduction

Tuberculosis (TB) remains a serious threat to global health and is one of the top 10 causes of death worldwide (WHO, 2019). In 2020, it was reported that 3.3% of new cases and 17.7% of previously treated cases were classified as multi-drug resistant (MDR) or rifampicin-resistant TB (RR-TB). For drug-susceptible TB infections, a regimen of first-line drugs (isoniazid, rifampicin, ethambutol and pyrazinamide) is administered for 6 months. Drug-resistant infections require treatments that are often longer in duration and more toxic. They are also more expensive, costing up to 25 times more than the regimen used to treat non-resistant infections (Marks et al., 2014).

The mycobacterial membrane protein large (MmpL) family of proteins, of which there are 13 members, is encoded by the *Mycobacterium tuberculosis* (*Mtb*) genome (Cole et al., 1998). Together, these proteins are responsible for the translocation of a diverse array of cell envelope lipids across the plasma membrane (Viljoen et al., 2017, Melly and Purdy, 2019). MmpL3 is the only essential MmpL; it is conserved across the *Mycobacterium* genus, and as such, is an attractive drug target (Cole et al., 1998, Deidda et al., 1998, Domenech et al., 2005, Protopopova et al., 2005, Stanley et al., 2012, La Rosa et al., 2012, Onajole et al., 2013, Rao et al., 2013, Remuiñán et al., 2013, Li et al., 2014b, Li et al., 2014a, Graham et al., 2018). Its primary role is the transport of trehalose-monomycolate (TMM), an essential component involved in cell envelope synthesis, and precursor to trehalose dimycolate (TDM), and cell wall-bound mycolic acids (Belisle et al., 1997, Grzegorzewicz et al., 2012, Tahlan et al., 2012). Depletion of MmpL3 causes an accumulation of TMM/TDM precursors and ultimately results in cell death through depletion of cell wall-bound mycolates (Degiacomi et al., 2017). Elucidation of the crystal structure of MmpL3 led to the prediction that it acts as a flippase and is capable of binding phosphatidylethanolamine (PE) and TMM (Su et al., 2019, Zhang et al., 2019).

Inhibitors of MmpL3 are numerous and structurally diverse; they include pyrroles, pyrazoles, benzimidazoles and derivatives of carboxamide and ethylenediamine (Protopopova et al., 2005, La Rosa et al., 2012, Stanley et al., 2012, Onajole et al., 2013, Remuiñán et al., 2013, Graham et al., 2018). Many of these inhibitors have been shown to act as multi-target agents, with secondary targets in *Mtb* as well as in other non-MmpL3-containing pathogens (Li et al., 2014a). For example SQ109, an ethylenediamine-based inhibitor of MmpL3 currently undergoing clinical trials, also inhibits a wealth of organisms including the Gram-negative bacterium *Helicobacter pylori* (Makobongo et al., 2013) and Gram-positive bacterium *Staphylococcus aureus*, the yeasts *Saccharomyces cerevisiae* and *Candida albicans*, and the malaria parasite *Plasmodium falciparum* (Li et al., 2014a). In addition, SQ109 shows activity against a range of trypanosomes, including the parasites *Trypanosoma cruzi* (Veiga-Santos et al., 2015), responsible for Chagas disease, and *Leishmania donovani* (Gil et al., 2020) and *L. mexicana* (García-García, Oldfield and Benaim, 2016), both of which cause leishmaniasis. In these species it acts as an uncoupler, disrupting mitochondrial membrane potential.

MmpL3 has consistently been identified as the target of hits emanating from whole-cell screens, through the generation of resistant mutants; this target identification method, however, is not always reliable and can lead to incorrect target assignment. The abundance of proposed MmpL3 inhibitors and their structural variety has led to significant debate surrounding their mechanisms of action, and whether they act through specific inhibition of MmpL3 or through indirect pathways (Viljoen, 2017). There are strong indications that MmpL5 and MmpL7, for example, can also act as efflux pumps (Pasca et al., 2005), with mutations conferring cross-resistance to several drugs, including azoles (Milano et al., 2009) and bedaquiline (Hartkoorn, Uplekar and Cole, 2014), concordant in their upregulation. This has led to speculation that spontaneous resistance mutations in MmpL3, could likewise be a resistance mechanism, rather than identifying MmpL3 as the mode of action (Li et al., 2014b, Cox et al., 2016). Recently, co-crystallisation of MmpL3 with three of the most widely studied MmpL3 inhibitors, namely SQ109, AU1235 and ICA38, demonstrated that all three drugs directly bind to the same binding pocket in the transmembrane region of MmpL3 (Zhang et al., 2019). Binding of these inhibitors results in a conformational change that disrupts the proton motive force and blocks substrate translocation, inhibiting MmpL3 function (Zhang, 2019). Molecular docking predicts that six other MmpL3 inhibitors, including BM212, bind to the same pocket (Zhang, 2019; Bolla, 2020).

Further to the more general modes of action of many of these inhibitors, such as dissipating the proton motive force, the energy force driving substrate transfer in MmpL3 and other transporters (Li et al., 2014a, Li et al., 2014b), an increasing number of MmpL3 inhibitors have been discovered to have additional targets. One such compound is SQ109, which also acts as an inhibitor of MenA and MenB in *Mtb*, two enzymes involved in the synthesis of the critical electron transport chain component, menaquinone (Li et al., 2014a, Berube et al., 2019). Another compound that has been described as an MmpL3 inhibitor, based on the generation of spontaneous resistant mutants, is tetrahydropyrazo[1,5-a]pyrimidine-3-carboxamide (THPP). Using bead immobilisation assays, EchA6 was determined as an another target for THPP, again demonstrating that the generation of resistance mutations in MmpL3 can distract from target identification (Cox et al., 2016).

Here we investigate the targets of the pyrrole derivative, BM212 (1,5-diaryl-2-methyl-3-(4-methylpiperazin-1-yl)methyl-pyrrole) and analogues from the BM212 series, known for their potent inhibitory effect against multidrug-resistant strains of *Mtb.* To determine alternative targets of BM212, which may have been masked by MmpL3 mutations, we applied a combination of chemo-profiling using bead-immobilised compounds, and whole-cell and biochemical assays. EthR2 (Rv0078) was identified by chemo-profiling as a protein that binds BM212 analogues. We demonstrated further the binding of BM212 to EthR2 through a tryptophan fluorescence assay, which showed significant quenching of tryptophan fluorescence upon addition of BM212.

## Materials and methods

### Chemical synthesis

#### Analytical methods

Reactions were carried out under nitrogen using dry solvents. All reagents were used as received from commercial suppliers. NMR data were recorded on a Bruker AVIII300, Bruker DPX400 or AVIII400 spectrometer (Supplementary NMR Data). Chemical shifts (*δ*) are quoted in ppm and coupling constants (*J*) are reported in Hz to one decimal place. Spectra were recorded in deuterated chloroform (unless otherwise indicated) and calibrated using residual solvent resonances (^1^H = 7.26 ppm; ^13^C = 77.16 ppm). The multiplicities of ^1^H NMR resonances are abbreviated as follows: s (singlet), d (doublet), t (triplet), q (quartet), m (multiplet). Data for ^1^H NMR spectra are reported as follows: chemical shift (multiplicity, coupling constant, number of protons); and for proton-decoupled ^13^C NMR spectra: chemical shift. 2-Dimensional homonuclear (^1^H–^1^H) and heteronuclear (^1^H–^13^C) NMR experiments were used to make unequivocal assignments. The progress of reactions was monitored by thin layer chromatography using Merck silica gel 60 F_254_ plates, which were visualised with UV light and subsequent staining using *p*-anisaldehyde, acidic potassium permanganate or ninhydrin.

LCMS analysis was conducted on an Agilent HPLC. Method: neutral with ammonium carbonate pH 7. (4.5 min chromatogram). Initial conditions: 70:30 ammonium carbonate:MeCN. The UV detection was an averaged signal from wavelength of 210 nm to 350 nm. Mass spectra were recorded on a Waters ZMD mass spectrometer using alternate-scan positive- and negative-mode electrospray ionisation (ES + ve and ES –ve), mass range 100 – 1200. In those compounds where significantly populated isotopes are present (Cl, Br), only the lower isotopomer is reported.

Microwave reactions were conducted using a Biotage Initiator microwave. The initial absorption was set as ‘high’ and 15 s of pre-stirring was applied before heating commenced. Flash column chromatography was carried out using Davisil 60 Å silica gel and the indicated solvent systems. Preparative HPLC separation was conducted on an Agilent 1200 or on an Agilent 1100, using either an X-Bridge C18 column (19 mm × 150 mm, i.d. 5 µm packing diameter) or an X-Bridge C18 column (30 mm × 150 mm, i.d. 5 µm packing diameter) at 35 °C. The solvents employed were: A = 0.1 M formic acid in water; B = 0.1 M formic acid in acetonitrile. The purification was run as a gradient (A:B) over either 20 min, with a flow rate of 17 mL/min (19 mm × 150 mm, i.d. 5 µm packing diameter) or 35 mL/min (30 mm × 150 mm, i.d. 5 µm packing diameter). The UV detection wavelengths were 210 nm and 350 nm. All final products were lyophilised prior to their use in any biological assay. The purity of final compounds was>95% by LCMS (HPLC: Acquity UPLC BEH C18 1.7u 3 × 50 mm, 35 °C. Method: ammonium acetate 25 mM + 10% acetonitrile at pH 6.6/acetonitrile. 0 – 0.2 min 100:0; 0.2 – 1.0 min 10:90; 1.0 – 1.8 min 10:90; 1.8 – 2.0 min 100:0. Flow: 0.8 mL/min. The UV detection wavelength was 254 nm and 210 nm.)

#### General synthetic procedures

**General Procedure A – Stetter reaction.** Triethylamine (2.0 eq) and 3-buten-2-one (1.2 eq) were added sequentially to a solution of the corresponding benzaldehyde (1.0 eq) and 3-ethyl-5-(2-hydroxyethyl)-4-methylthiazolium bromide (0.2 eq) in anhydrous acetonitrile (0.325 M) in a Schlenk flask under nitrogen. The flask was maintained under a nitrogen atmosphere and heated at 80 °C. After 24 h, the mixture was quenched with 2 M hydrochloric acid, extracted with EtOAc (×3), and washed sequentially with saturated NaHCO_3_ solution and brine. The organic layers were combined, dried with Na_2_SO_4_ and concentrated under reduced pressure. The residue was purified by column chromatography (cyclohexane/EtOAc 8:1 to 3:1, gradient) to afford the desired product.

**General Procedure B - Paal-Knorr reaction.** Camphorsulfonic acid (0.25 eq) was added to a solution of the 1,4-diketone (1.0 eq) and the aniline (1.2 eq) in anhydrous methanol (0.15 M) in a microwave vial under nitrogen. The mixture was heated at 150 °C for 20 min in a microwave reactor. The solvent was then evaporated under reduced pressure and the crude product was directly purified by column chromatography (cyclohexane/EtOAc 8:1 to 3:1, gradient) to afford the desired product.

**General Procedure C - Mannich Reaction.** A solution of the secondary amine (1.0 eq) and formaldehyde (37%, 1.0 eq) in acetic acid (1 M) was added at room temperature and in one portion to a solution of 1,2,5-trisubstituted pyrrole (1.0 eq) in anhydrous acetonitrile (0.125 M) under a nitrogen atmosphere. After 15 h, the reaction was quenched with sodium hydroxide solution (10%), extracted with EtOAc (×3), and washed sequentially with water (×3) and brine. The organic layers were combined, dried with Na_2_SO_4,_ and concentrated under reduced pressure. The crude product was purified by column chromatography (cyclohexane/EtOAc 4:1 to 2:1, gradient) to afford the desired product.

**General Procedure D - Mitsunobu reaction.** A solution of (cyanomethylene)tributylphosphorane (1 M in toluene, 1.5 eq) was added under nitrogen to a solution of phenol derivative (1.0 eq) and benzyl (3-hydroxypropyl)carbamate (1.1 eq) in anhydrous toluene (0.4 M) in a microwave vial. The reaction vial was placed in a microwave reactor and heated at 140 °C for 30 min. The reaction solvent was then evaporated under reduced pressure and the crude product purified by column chromatography (cyclohexane/EtOAc 4:1 to 1:4, gradient) to afford the desired product.

**General Procedure E - Hydrogenolysis.** Nitrogen gas was bubbled through a solution of Cbz-protected compound (1.0 eq) in MeOH (0.15 M). Palladium on carbon (5 eq) was subsequently added. The atmosphere was displaced and maintained with hydrogen under atmospheric pressure. After 15 min, the palladium residues were filtered off, the solvent was removed from the filtrate under reduced pressure and the crude product was purified by preparative HPLC to afford the desired product.

##### Chemical synthesis of BM212

As outlined in Supplementary Scheme 1, 1-(4-chlorophenyl)pentane-1,4-dione was prepared following general procedure A using 4-chlorobenzaldehyde (2.81 g, 20.0 mmol). The 1,4-dione product was isolated as a white solid (3.65 g, 87%). ^1^H NMR (300 MHz, CDCl_3_) *δ*_H_ 7.97 – 7.84 (m, 2H), 7.46 – 7.35 (m, 2H), 3.26 – 3.19 (m, 2H), 2.92 – 2.77 (m, 2H), 2.25 (s, 3H);^13^C NMR (101 MHz, CDCl_3_) *δ*_C_ 207.2, 197.3, 139.6, 135.0, 129.5, 128.9, 37.0, 32.3, 30.1; *m*/*z* (EI) C_11_H_11_ClO_2_, found [M]^+^ 210.1. Spectroscopic data were in agreement with those reported in the literature (Chochois et al., 2006).

1,2-Bis(4-chlorophenyl)-5-methyl-1*H*-pyrrole was prepared following general procedure B using 1-(4-chlorophenyl)pentane-1,4-dione (666 mg, 3.16 mmol) and 4-chloroaniline (484 mg, 3.79 mmol). The resulting pyrrole product, (1,2-bis(4-chlorophenyl)-5-methyl-1*H*-pyrrole), was isolated as a yellowish solid (955 mg, 95%). ^1^H NMR (400 MHz, CDCl_3_) *δ*_H_ 7.39 – 7.32 (m, 2H), 7.17 – 7.10 (m, 2H), 7.10 – 7.05 (m, 2H), 6.99 – 6.93 (m, 2H), 6.34 (d, *J* = 3.5 Hz, 1H), 6.09 (dq, *J* = 3.5, 0.6 Hz, 1H), 2.13 (d, *J* = 0.6 Hz, 3H); ^13^C NMR (101 MHz, CDCl_3_) *δ*_C_ 137.7, 133.5, 133.0, 132.1, 131.71, 131.68 129.6, 129.4, 128.9, 128.3, 109.4, 108.1, 13.3; *m*/*z* (EI) C_17_H_13_Cl_2_N, found [M]^+^ 302.1. ^1^H NMR Spectroscopic data consistent with those reported in the literature (Kamal et al., 2016).

1-((1,5-Bis(4-chlorophenyl)-2-methyl-1*H*-pyrrol-3-yl)methyl)-4-methylpiperazine was prepared following general procedure C using 1,2-bis(4-chlorophenyl)-5-methyl-1*H*-pyrrole (50 mg, 0.16 mmol) and *N-*methylpiperazine (18 μL, 0.16 mmol). 1,2,4,5-Tetrasubstituted pyrrole product [BM212, 1-((1,5-bis(4-chlorophenyl)-2-methyl-1*H*-pyrrol-3-yl)methyl)-4-methylpiperazine] was isolated as a white solid (45 mg, 66%).^1^H NMR (400 MHz, CDCl_3_) *δ*_H_ 7.37 – 7.30 (m, 2H), 7.13 – 7.08 (m, 2H), 7.07 – 7.01 (m, 2H), 6.96 – 6.90 (m, 2H), 6.35 (s, 1H), 3.47 (s, 2H), 3.04 – 2.38 (br m, 8H), 2.30 (s, 3H), 2.06 (s, 3H). ^13^C NMR (101 MHz, CDCl_3_) *δ*_C_ 137.7, 133.4, 131.9, 131.6, 131.5, 130.2, 129.7, 129.4, 128.8, 128.3, 116.7, 111.7, 55.0, 54.3, 52.6, 46.0, 11.2; *m*/*z* (EI) C_23_H_25_Cl_2_N_3_, found [M]^+^ 414.2. Spectroscopic data were in agreement with those reported in the literature (More et al., 2016).

##### Chemical synthesis of GSK074A

As outlined in Supplementary Scheme 2, 1-(4-isopropylphenyl)pentane-1,4-dione was prepared following general procedure A using 4-isopropylbenzaldehyde (1.48 g, 10.0 mmol). The 1,4-dione product was isolated as a yellow oil (1.1 g, 50%). ^1^H NMR (400 MHz, CDCl_3_) *δ*_H_ 7.95 – 7.89 (m, 2H), 7.33 – 7.28 (m, 2H), 3.28 – 3.22 (m, 2H), 3.07 – 2.90 (m, 1H), 2.90 – 2.83 (m, 2H), 2.25 (s, 3H), 1.26 (d, *J* = 6.9 Hz, 6H); ^13^C NMR (101 MHz, CDCl_3_) *δ*_C_ 207.5, 198.2, 154.7, 134.5, 128.3, 126.7, 37.1, 34.3, 32.3, 30.1, 23.7; *m*/*z* (EI) C_14_H_18_O_2_, found [M]^+^ 218.3. ^1^H NMR Spectroscopic data were in agreement with those reported in the literature (Biava et al., 2008).

1-(4-Fluorophenyl)-2-(4-isopropylphenyl)-5-methyl-1*H*-pyrrole was prepared following general procedure B using 1-(4-isopropylphenyl)pentane-1,4-dione (1.10 g, 5.0 mmol) and 4-fluoroaniline (574 μL, 6*.*0 mmol). The 1,2,5-trisubstituted pyrrole product was isolated as a yellow oil that solidified upon standing (1.25 g, 85%). ^1^H NMR (400 MHz, CDCl_3_) *δ*_H_ 7.19 – 7.11 (m, 2H), 7.10 – 6.95 (m, 6H), 6.33 (d, *J* = 3.4 Hz, 1H), 6.09 (dd, *J* = 3.4, 0.6 Hz, 1H), 2.83 (heptet, *J* = 6.9 Hz, 1H), 2.13 (d, *J* = 0.6 Hz, 3H), 1.21 (d, *J* = 6.9 Hz, 6H).^13^C NMR (101 MHz, CDCl_3_) *δ*_C_ 161.6 (d, *J* = 247.1 Hz), 146.4, 135.6, 134.4, 131.4, 130.8, 130.1 (d, *J* = 8.4 Hz), 127.7, 126.1, 115.9 (d, *J* = 22.7 Hz), 108.3, 107.5, 33.6, 23.9, 13.3; *m*/*z* (ES + ) C_20_H_20_FN, found [M + H]^+^ 294.2. ^1^H NMR Spectroscopic data were in agreement with those reported in the literature (Biava et al., 2008).

1-((1-(4-Fluorophenyl)-5-(4-isopropylphenyl)-2-methyl-1*H*-pyrrol-3-yl)methyl)-4-methylpiperazine (GSK074A) was prepared following general procedure C using 1-(4-fluorophenyl)-2-(4-isopropylphenyl)-5-methyl-1*H*-pyrrole (160 mg, 0.5 mmol) and *N-*methylpiperazine (60 μL, 0.5 mmol). The 1,2,4,5-tetrasubstituted pyrrole product (GSK074A) was isolated as an off-white solid (99 mg, 45%). ^1^H NMR (400 MHz, CDCl_3_) *δ*_H_ 7.29 – 6.90 (m, 8H), 6.32 (s, 1H), 3.47 (s, 2H), 2.80 (heptet, *J* = 6.9 Hz, 1H), 2.73 – 2.34 (m, 8H), 2.29 (s, 3H), 2.05 (s, 3H), 1.18 (d, *J* = 6.9 Hz, 6H). ^13^C NMR (101 MHz, CDCl_3_) *δ*_C_ 161.6 (d, *J* = 247.1 Hz), 146.3, 135.7, 135.7, 133.3, 130.6, 130.2 (d, *J* = 8.5 Hz), 129.5, 127.5, 126.1, 116.2, 115.8 (d, *J* = 22.6 Hz), 110.76, 110.75, 55.2, 54.4, 52.7, 46.0, 33.6, 23.9, 11.2; *m*/*z* (ES + ) C_26_H_32_FN_3_, found [M + H]^+^ 406.4.

##### Chemical synthesis of GSK303A

As outlined in Supplementary Scheme 3, 2-(4-chlorophenyl)-5-methyl-1*-*(4-methylphenyl)*-*1*H*-pyrrole was prepared following general procedure B using 1-(4-chlorophenyl)pentane-1,4-dione (200 mg, 0.9 mmol) and *p*-toluidine (115 μL, 1.1 mmol). The pyrrole product was isolated as a yellow solid (194 mg, 73%). ^1^H NMR (400 MHz, CDCl_3_) *δ*_H_ 7.22 – 7.15 (m, 2H), 7.14 – 7.08 (m, 2H), 7.06 – 7.02 (m, 2H), 7.01 – 6.97 (m, 2H), 6.36 (d, *J* = 3.5 Hz, 1H), 6.10 (dd, *J* = 3.5, 0.7 Hz, 1H), 2.40 (s, 3H), 2.14 (d, *J* = 0.7 Hz, 3H). ^1^H NMR Spectroscopic data were in agreement with those reported in the literature (Biava et al., 2008).

4-((5-(4-Chlorophenyl)-2-methyl-1-(4-methylphenyl)-1*H*-pyrrol-3-yl)methyl)morpholine) (GSK303A) was prepared following general procedure C using 2-(4-chlorophenyl)-5-methyl-1-(4-methylphenyl)-1*H*-pyrrole (194 mg, 0.7 mmol) and morpholine (60 μL, 0.7 mmol). The 1,2,4,5-tetrasubstituted pyrrole product (GSK303A) was isolated as an off-white solid (150 mg, 57%). ^1^H NMR (400 MHz, CDCl_3_) *δ*_H_ 7.19 – 7.15 (m, 2H), 7.11 – 7.05 (m, 2H), 7.03 – 6.99 (m, 2H), 6.98 – 6.94 (m, 2H), 6.35 (s, 1H), 3.79 – 3.69 (m, 4H), 3.43 (s, 2H), 2.52 (br s, 4H), 2.38 (s, 3H), 2.06 (s, 3H); ^13^C NMR (101 MHz, CDCl_3_) *δ*_C_ 137.5, 136.6, 131.90, 131.88, 131.3, 130.4, 129.8, 128.6, 128.3, 128.1, 116.2, 111.0, 67.1, 55.1, 53.5, 21.2, 11.2; *m*/*z* (ES + ) C_23_H_25_ClN_2_O, found [M + H]^+^ 381.3. Data were in agreement with those reported in the literature (Poce et al., 2013).

##### Chemical synthesis of GSK569A

As outlined in Supplementary Scheme 4, 4-(2-(4-isopropylphenyl)-5-methyl-1*H*-pyrrol-1-yl)phenol was prepared following general procedure B using 1-(4-isopropylphenyl)pentane-1,4-dione (437 mg, 2.0 mmol) and 4-aminophenol (261 mg, 2.4 mmol). The 1,2,5-trisubstituted pyrrole product was isolated as a purple oil (563 mg, 97%). ^1^H NMR (400 MHz, CDCl_3_) *δ*_H_ 7.09 – 7.01 (m, 2H), 7.00 (s, 4H), 6.84 – 6.78 (m, 2H), 6.30 (d, *J* = 3.4 Hz, 1H), 6.06 (dd, *J* = 3.4, 0.7 Hz, 1H), 4.76 (s, 1H), 2.81 (heptet, *J* = 6.9 Hz, 1H), 2.11 (d, *J* = 0.7 Hz, 3H), 1.19 (d, *J* = 6.9 Hz, 6H); ^13^C NMR (101 MHz, CDCl_3_) *δ*_C_ 154.6, 146.2, 134.3, 132.6, 131.5, 131.0, 129.7, 127.6, 126.0, 115.7, 107.9, 107.0, 33.6, 23.9, 13.3; *m*/*z* (ES + ) C_20_H_21_NO, found [M + H]^+^ 291.9.

Benzyl (3-(4-(2-(4-isopropylphenyl)-5-methyl-1*H*-pyrrol-1-yl)phenoxy)propyl)carbamate was prepared following general procedure D using 4-(2-(4-isopropylphenyl)-5-methyl-1*H*-pyrrol-1-yl)phenol (250 mg, 0.86 mmol) and benzyl (3-hydroxypropyl)carbamate (Miller et al., 2004) (198 mg, 0.94 mmol). The carbamate product was isolated as a yellow oil (402 mg, 97%). ^1^H NMR (400 MHz, CDCl_3_) *δ*_H_ 7.40 – 7.26 (m, 5H), 7.11 – 7.00 (m, 2H), 6.99 (s, 4H), 6.88 – 6.79 (m, 2H), 6.30 (d, *J* = 3.4 Hz, 1H), 6.05 (dd, *J* = 3.4, 0.8 Hz, 1H), 5.11 (s, 2H), 4.99 (s, 1H), 4.03 (t, *J* = 5.8 Hz, 2H), 3.44 (app q, *J* = 6.4 Hz, 2H), 2.87 – 2.74 (m, 1H), 2.10 (d, *J* = 0.8 Hz, 3H), 2.07 – 1.97 (m, 2H), 1.18 (d, *J* = 6.9 Hz, 6H); ^13^C NMR (101 MHz, CDCl_3_) *δ*_C_ 157.7, 156.4, 146.1, 134.3, 132.6, 131.6, 131.0, 129.7, 129.5, 128.6, 128.2, 127.6, 126.0, 114.6, 107.9, 107.0, 66.7, 66.0, 38.6, 33.6, 29.5, 23.9, 13.3, 1 × aromatic C not observed, possible resonance overlap; *m*/*z* (ES + ) C_31_H_34_N_2_O_3_, found [M + H]^+^ 483.2.

Benzyl (3-(4-(5-(4-isopropylphenyl)-2-methyl-3-(morpholinomethyl)-1*H*-pyrrol-1-yl)phenoxy)propyl)-carbamate was prepared following general procedure C using benzyl (3-(4-(2-(4-isopropylphenyl)-5-methyl-1*H*-pyrrol-1-yl)phenoxy)propyl) carbamate (400 mg, 0.82 mmol) and morpholine (75 μL, 0.82 mmol). The morpholine product was isolated as an orange oil (250 mg, 50%). ^1^H NMR (400 MHz, CDCl_3_) *δ*_H_ 7.39 – 7.28 (m, 5H), 7.12 – 7.03 (m, 2H), 6.99 (s, 4H), 6.87 – 6.83 (m, 2H), 6.33 (s, 1H), 5.11 (s, 3H), 4.03 (t, *J* = 5.8 Hz, 2H), 3.77 – 3.71 (m, 4H), 3.49 – 3.37 (m, 4H), 2.80 (heptet, *J* = 6.9 Hz, 1H), 2.53 (s, 4H), 2.08 – 1.95 (m, 5H), 1.18 (d, *J* = 6.9 Hz, 6H); ^13^C NMR (101 MHz, CDCl_3_) *δ*_C_ 157.7, 156.5, 146.1, 136.6, 133.2, 132.7, 130.8, 129.8, 129.6, 128.6, 128.2, 127.4, 126.1, 115.7, 114.6, 110.3, 67.1, 66.7, 65.9, 55.1, 53.5, 38.6, 33.6, 29.5, 23.9, 11.2, 1 × aromatic C not observed, possible resonance overlap or not well resolved; *m*/*z* (ES-) C_36_H_43_N_3_O_4_, found [M – H]^+^ 580.4.

3-(4-(5-(4-Isopropylphenyl)-2-methyl-3-(morpholinomethyl)-1*H*-pyrrol-1-yl)phenoxy)propan-1-amine (GSK569A) was prepared following general procedure E using benzyl (3-(4-(5-(4-isopropylphenyl)-2-methyl-3-(morpholinomethyl)-1*H*-pyrrol-1-yl)phenoxy)propyl)carbamate (250 mg, 0.43 mmol). The primary amine product (GSK569A) was isolated as an off-white solid (156 mg, 84%). %). ^1^H NMR (400 MHz, CDCl_3_) *δ*_H_ 7.08 – 7.02 (m, 2H), 6.98 (s, 4H), 6.92 – 6.84 (m, 2H), 6.31 (s, 1H), 4.05 (t, *J* = 6.1 Hz, 2H), 3.78 – 3.69 (m, 4H), 3.44 (s, 2H), 2.93 (t, *J* = 6.8 Hz, 2H), 2.85 – 2.74 (m, 1H), 2.52 (s, 4H), 2.04 (s, 3H), 1.95 (app pentet, *J* = 6.4 Hz, 2H), 1.18 (d, *J* = 6.9 Hz, 6H), NH_2_ not observed. ^13^C NMR (101 MHz, CDCl_3_) *δ*_C_ 158.0, 146.0, 133.2, 132.5, 130.8, 129.8, 129.6, 127.4, 126.0, 115.6, 114.6, 110.2, 67.1, 66.1, 55.1, 53.4, 39.2, 33.6, 33.0, 23.9, 11.1; *m*/*z* (ES + ) C_28_H_37_N_3_O_2_, found [MH – morpholine]^+^ 361.3.

##### Chemical synthesis of GSK574A

As outlined in Supplementary Scheme 5, 1-(4-hydroxyphenyl)pentane-1,4-dione was prepared following general procedure A, heating at 80 °C but for 72 h using 4-hydroxybenzaldehyde (2.44 g, 20.0 mmol). The 1,4-dione product was isolated as a yellowish oil that solidified upon standing (702 mg, 18%). ^1^H NMR (400 MHz, CDCl_3_) *δ*_H_ 7.86 – 7.79 (m, 2H), 6.87 – 6.81 (m, 2H), 3.18 (t, *J* = 6.2 Hz, 2H), 2.83 (t, *J* = 6.2 Hz, 2H), 2.22 (s, 3H), OH not observed; ^13^C NMR (101 MHz, CDCl_3_) *δ*_C_ 208.9, 197.7, 161.8, 130.6, 128.7, 115.5, 37.2, 32.0, 30.1. Data were in agreement with those reported in the literature (Esumi et al., 2016).

4-(1-(4-Fluorophenyl)-5-methyl-1*H*-pyrrol-2-yl)phenol was prepared following general procedure B using 1-(4-hydroxyphenyl)pentane-1,4-dione (702 mg, 3.7 mmol) and 4-fluoroaniline (420 μL, 4.4 mmol). The pyrrole product was isolated as a yellowish oil that solidified upon standing (436 mg, 45%). ^1^H NMR (400 MHz, CDCl_3_) *δ*_H_ 7.15 – 7.08 (m, 2H), 7.08 – 6.99 (m, 2H), 6.96 – 6.88 (m, 2H), 6.65 – 6.60 (m, 2H), 6.25 (d, *J* = 3.4 Hz, 1H), 6.07 (dd, *J* = 3.4, 0.7 Hz, 1H), 4.69 (br s, 1H), 2.12 (d, *J* = 0.7 Hz, 3H); ^13^C NMR (101 MHz, CDCl_3_) *δ*_C_ 161.6 (d, *J* = 247.3 Hz), 153.8, 135.4 (d, *J* 4.0 Hz), 134.1, 131.0, 130.1 (d, *J* = 8.5 Hz), 129.5, 126.3, 115.9 (d, *J* = 22.6 Hz), 115.0, 107.8, 107.3, 13.3; *m*/*z* (ES + ) C_17_H_14_FNO, found [M + H]^+^ 268.4.

Benzyl (3-(4-(1-(4-fluorophenyl)-5-methyl-1*H*-pyrrol-2-yl)phenoxy)propyl)carbamate was prepared following general procedure D using 4-(1-(4-fluorophenyl)-5-methyl-1*H*-pyrrol-2-yl)phenol (420 mg, 1.57 mmol) and benzyl (3-hydroxypropyl)carbamate (Miller et al., 2004) (345 mg, 1.65 mmol). The carbamate product was isolated as a yellowish oil (500 mg, 69%). ^1^H NMR (400 MHz, CDCl_3_) *δ*_H_ 7.39 – 7.29 (m, 5H), 7.14 – 7.08 (m, 2H), 7.08 – 7.01 (m, 2H), 6.99 – 6.93 (m, 2H), 6.68 (d, *J* = 8.7 Hz, 2H), 6.25 (d, *J* = 3.4 Hz, 1H), 6.06 (dd, *J* = 3.4, 0.6 Hz, 1H), 5.09 (s, 2H), 4.97 (br s, 1H), 3.95 (t, *J* = 5.8 Hz, 2H), 3.38 (app. q, *J* = 6.3 Hz, 2H), 2.12 (d, *J* = 0.6 Hz, 3H), 2.00 – 1.92 (m, 2H); ^13^C NMR (101 MHz, CDCl_3_) *δ*_C_ 161.6 (d, *J* = 247.2 Hz), 157.0, 156.4, 135.4, 134.1, 131.0, 130.0 (d, *J* = 8.5 Hz), 129.2, 128.5, 128.1, 126.3, 115.9 (d, *J* = 22.6 Hz), 114.1, 107.8, 107.4, 66.7, 65.7, 38.7, 29.4, 13.31, 1 × aromatic C and 1 × aromatic CH not observed, possible resonance overlap or not well resolved; *m*/*z* (ES + ) C_28_H_27_FN_2_O_3_, found [M + H]^+^ 459.2.

Benzyl (3-(4-(1-(4-fluorophenyl)-5-methyl-4-(morpholinomethyl)-1*H*-pyrrol-2-yl)phenoxy)propyl) carbamate was prepared following general procedure C using benzyl (3-(4-(1-(4-fluorophenyl)-5-methyl-1*H*-pyrrol-2-yl)phenoxy)propyl) carbamate (500 mg, 1.1 mmol) and morpholine (95 μL, 1.1 mmol). The morpholine product was isolated as an orange oil (193 mg, 32%). ^1^H NMR (400 MHz, CDCl_3_) *δ*_H_ 7.37 – 7.27 (m, 5H), 7.12 – 7.00 (m, 4H), 6.97 – 6.91 (m, 2H), 6.66 (d, *J* = 8.7 Hz, 2H), 6.26 (s, 1H), 5.09 (s, 2H), 4.99 (br s, 1H), 3.95 (t, *J* = 5.8 Hz, 2H), 3.76 – 3.72 (m, 4H), 3.44 (s, 2H), 3.41 – 3.33 (m, 2H), 2.53 (br s, 4H), 2.06 (s, 3H), 2.00 – 1.92 (m, 2H); ^13^C NMR (101 MHz, CDCl_3_) *δ*_C_ 161.6 (d, *J* 247.5 Hz), 157.0, 156.4, 135.5, 133.1, 130.2 (d, *J* 8.1 Hz), 129.2, 129.1, 128.5, 128.1, 126.1, 115.9 (d, *J*, 22.2 Hz), 114.1, 110.1, 67.1, 66.7, 65.7, 55.0, 53.5, 38.7, 29.4, 11.2, 2 × aromatic Cs not observed and 1 × aromatic CH not accounted for, probable resonance overlap; *m*/*z* (ES + ) C_33_H_36_FN_3_O_4_, found [MH – morpholine]^+^ 471.2.

3-(4-(1-(4-Fluorophenyl)-5-methyl-4-(morpholinomethyl)-1*H*-pyrrol-2-yl)phenoxy)propan-1-amine (GSK574A) was prepared following general procedure E using benzyl (3-(4-(1-(4-fluorophenyl)-5-methyl-4-(morpholinomethyl)-1*H*-pyrrol-2-yl)phenoxy)propyl)carbamate (193 mg, 0.34 mmol). The amine product (GSK574A) was isolated as a colourless oil (65 mg, 44%). ^1^H NMR (400 MHz, CDCl_3_) *δ*_H_ 7.14 – 6.99 (m, 4H), 6.96 – 6.88 (m, 2H), 6.71 – 6.65 (m, 2H), 6.26 (s, 1H), 3.97 (t, *J* = 6.4 Hz, 2H), 3.75 – 3.71 (m, 4H), 3.43 (s, 2H), 2.87 (t, *J* = 6.4 Hz, 2H), 2.52 (br., 4H), 2.06 (s, 3H), 1.88 (app. pentet, *J* = 6.4 Hz, 2H), NH_2_ not observed; ^13^C NMR (101 MHz, CDCl_3_) *δ*_C_ 161.5 (d, *J* 248.5 Hz), 157.2, 135.6, 133.1, 130.2 (d, *J* 9.1 Hz), 129.1, 125.8, 115.9 (d, *J*, 22.2 Hz), 114.1, 110.0, 67.1, 65.8, 55.1, 53.5, 39.3, 33.0, 11.1, 2 × aromatic Cs not observed; *m*/*z* (ES + ) C_25_H_30_FN_3_O_2_, found [MH – morpholine]^+^ 337.2.

A solution of benzyl chloroformate (2.84 mL, 20 mmol) in CH_2_Cl_2_ (10 mL) was added over 2 min to a solution of 3-aminopropan-1-ol (3.82 mL, 50 mmol) in CH_2_Cl_2_ (40 mL) at 0 °C. The mixture was allowed to warm to RT. After 3 h, the mixture was diluted with CH_2_Cl_2_ (25 mL) and washed sequentially with saturated NH_4_Cl solution (3 × 40 mL) and brine (40 mL), dried over Na_2_SO_4_ and concentrated under reduced pressure. The residue was purified by flash column chromatography (gradient: EtOAc/cyclohexane 1:3 to 1:1) to afford benzyl (3-hydroxypropyl)carbamate as a white solid (3.27 g, 78%). ^1^H NMR (400 MHz, CDCl_3_) *δ*_H_ 7.40 – 7.28 (m, 5H), 5.11 (s, 2H), 5.02 (br s, 1H), 3.68 (app. q, *J* = 5.7 Hz, 2H), 3.36 (app. q, *J* = 6.3 Hz, 2H), 2.52 (br s, 1H), 1.75 – 1.66 (m, 2H). ^1^H NMR spectroscopic data in accordance with those reported in the literature (Miller et al., 2004).

### Chemical proteomics studies

#### Chemical proteomics

Chemical proteomics experiments were performed as previously described (Bantscheff et al., 2011). Briefly, NHS-activated Sepharose beads were derivatised with compound GSK569A at 0.3 mM and 1 mM, and GSK574A at 1 mM, and washed and equilibrated in lysis buffer (50 mM Tris-HCl, pH 7.4, 0.02% – 0.4% Igepal-CA630, 1.5 mM MgCl_2_, 5% glycerol, 150 mM NaCl, 25 mM NaF, 1 mM Na_3_VO_4_, 1 mM DTT, and one complete EDTA-free protease inhibitor tablet (Roche) *per* 25 mL). The functionalised beads were incubated at 4 °C for 1 h with 0.1 mL (0.25 mg) *Mycobacterium bovis* BCG extract, which was pre-incubated with either test compounds GSK074A or GSK303A, or DMSO (vehicle control). *M. bovis* BCG extract was generated as described in Abrahams et al. (2016).

The experimental set up was such that 10 samples were multiplexed (TMT 10-plex, (Werner et al., 2014)) to generate values for the affinity of the beads to the bound proteins (“depletion” values, 4 samples) and to generate IC_50_ values (6 samples) in a single experiment. Samples 1 and 2 were the vehicle control, samples 3 and 4 were processed in the same way, but while the beads were discarded after the first incubation step, the extract was incubated with fresh beads to measure how much protein (depleted from the extract by first bead-binding) could rebind to fresh beads (Eberl et al., 2019). Apparent dissociation constants were determined by considering the protein depletion by the beads (Bantscheff et al., 2011). Samples 5 – 10 were used to generate IC_50_ values by adding compound over a range of concentrations (40 µM, 1:3 dilutions). Beads were transferred to filter plates (Durapore (PVDF membrane, Merck Millipore), washed extensively with lysis buffer and eluted with SDS sample buffer. Proteins were digested with trypsin following a modified single-pot solid-phase sample preparation (SP3) protocol (Hughes et al., 2014, Moggridge et al., 2018). Peptides were labelled with isobaric mass tags (TMT10, Thermo Fisher Scientific, Waltham, MA) using the 10-plex TMT reagents (Werner et al., 2012, 2014) and labelled peptide extracts were combined to a single sample per experiment, lyophilised and subjected to LC-MS analysis. LC-MS/MS measurements using Q Exactive Orbitrap or Orbitrap Fusion Lumos mass spectrometers (Thermo Fisher Scientific) were performed as described (Penzo et al., 2019, Sridharan et al., 2019). For peptide and protein identification, see Wilson et al., 2017, Sridharan et al., 2019.

Unless stated otherwise, we accepted protein identifications as follows: (i) For single spectrum to sequence assignments, we required this assignment to be the best match and a minimum Mascot score of 31 and a 10 × difference of this assignment over the next best assignment. Based on these criteria, the decoy search results indicated < 1% false discovery rate (FDR). (ii) For multiple spectrum to sequence assignments and using the same parameters, the decoy search results indicated < 0.1% FDR. Quantified proteins were required to contain at least 2 unique peptide matches. FDR for quantified proteins was < 0.1%. Experimental design and raw data tables for the chemoproteomics experiments can be found in the Supplementary Tables 1 and 2.

### Biochemical studies

#### Overexpression and purification of EthR2

pET28a-*ethR2* (*rv0078*) was transformed into *E. coli* BL21 (DE3) and cells were grown in 1 L of terrific broth. 1 mM isopropyl β-D-1-thiogalactopyranoside was added when cultures reached an OD_600_ of 0.8, and cultures were incubated at 16 °C for a further 12 h. Cultures were pelleted by centrifugation and resuspended in lysis buffer (50 mM NaH_2_PO_4_, 300 mM NaCl and 10 mM imidazole). Cells were lysed by sonication on ice for 10 min (20 s on, with 40 s cooling) and the insoluble material pelleted by centrifugation at 27,000 × g for 40 min at 4 °C. EthR2 was purified by nickel affinity chromatography using a HiTrap column (Qiagen). EthR2 was eluted at 50 mM imidazole and dialysed overnight into buffer A (25 mM Tris, 300 mM NaCl and 10% glycerol).

#### Tryptophan binding assay

Tryptophan residues carry intrinsic fluorescence; excitation of these hydrophobic amino acids at 295 nm results in an emission spectrum at 355 nm. Quenching of tryptophan fluorescence can occur due to protein folding, conformational changes to proteins, or binding to ligands. Fluorescence spectroscopy was used to measure the intrinsic tryptophan fluorescence of EthR2 in the presence of BM212. BM212 was added at increasing concentrations (1.25 µM, 2.5 µM, 5 µM, 10 µM, 20 µM and 40 µM) to a total reaction volume of 400 µL buffer, with a final concentration of 6 µM EthR2. The reaction mix was stirred for 2 min before measuring fluorescence emission at a range of 300 nm – 400 nm, with an excitation of 295 nm.

### Microbiological studies

#### Growth conditions for mycobacterial species

Mycobacterial species were grown in Middlebrook 7H9 broth containing 10% albumin dextrose catalase (ADC, Middlebrook), 0.05% Tween 80 and 0.2% glycerol, and cultures were grown in a static incubator at 37 °C. Unless otherwise stated, kanamycin was added for the maintenance of plasmids, at 50 µg/mL for *E. coli* and 25 µg/mL for *M. bovis* BCG.

#### Construction of recombinant mycobacterial strains

*Rv0077c* and *rv0078* were amplified by polymerase chain reaction using the primers in Table 1. The amplified fragments were digested with BamHI and HindIII and ligated into pMV261 and NdeI and HindIII for pET28a. Constructs were sequenced prior to transformation into *E. coli* and *M. bovis* BCG.

**Table 1:** Oligonucleotides used in gene and protein overexpression studies. Transformation of *E. coli* with pET28a for the overexpression and purification of EthR2 was carried out by heat shock and cells were plated on LB agar containing 50 µg/mL kanamycin. Transformation of pMV261 into *M. bovis* BCG for overexpression studies was carried out by electroporation, and cells were spread onto 7H11 plates supplemented with 25 µg/mL kanamycin.

Vector	Gene	Direction	Sequence
pMV261	*rv0077c*	Forward	CTAGCTAGGGATCCAATGTCGACGATCGACATTAG
		Reverse	CTAGCTAGAAGCTTCTACGTGCGCACCGCGACCG
	*rv0078*	Forward	CTAGCTAGGGATCCAATGGAAATCAAGAGACGCAC
		Reverse	CTAGCTAGAAGCTTCTAGCCGTTAAGCATCCCGTC
pET28a	*rv0078*	Forward	CTAGCTAGCATATGGAAATCAAGAGACGCACCCAG
		Reverse	CTAGCTAGAAGCTTCTAGCCGTTAAGCATCCCGTC

#### Determination of MICs

Liquid MICs were determined using 96-well Greiner black clear bottom plates, containing 100 µL liquid culture per well without kanamycin and a combination of BM212 and / or ETA dissolved in DMSO, with concentrations ranging from 0.5 µg/mL to 4 µg/mL and 0.125 µg/mL to 32 µg/mL respectively. Plates were set up with a starting optical density at 600 nm (OD_600_) of 0.1 and read using a spectrophotometer after 6 days. Data were normalised by subtracting the mean of negative controls and calculating percentage survival, where cultures grown without drug represented 100% survival. Dose-response curves were generated with x-axis values (drug concentrations) converted to logarithmic scale.

#### Analysis of drug interactions

Analysis of data from liquid MIC plates was carried out using the fractional inhibitory concentration (FIC) index to determine the level of synergy, antagonism or additivity/indifference. The OD_600_ after six days was used to determine drug interactions. OD readings were normalised to give a fractional response. The FIC index was calculated using equation 1, and interpreted as: ∑FIC < 0.5 = synergy; ∑FIC 0.5–4 = additive or indifference; ∑FIC > 4 = antagonism.

$$\sum FIC={\mathrm{FIC}}_{A}+{\mathrm{FIC}}_{B}=\frac{A}{{\mathrm{MIC}}_{A}}+\frac{B}{{\mathrm{MIC}}_{B}}$$

Equation 1 Fractional inhibitory concentration index, where A and B are the concentrations of the drugs A and B in combination, and MICA and MICB are the MICs of drug A and drug B when applied separately.

## Results

### Chemical proteomics studies

**Fig. 1:**
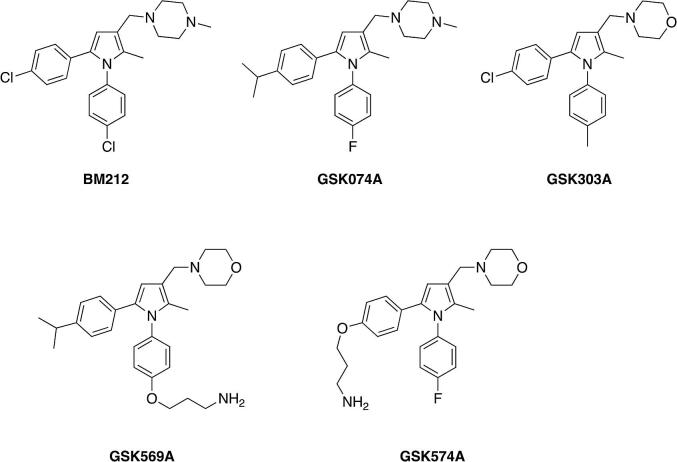
BM212 and analogues used in this study.

**Scheme 1:**
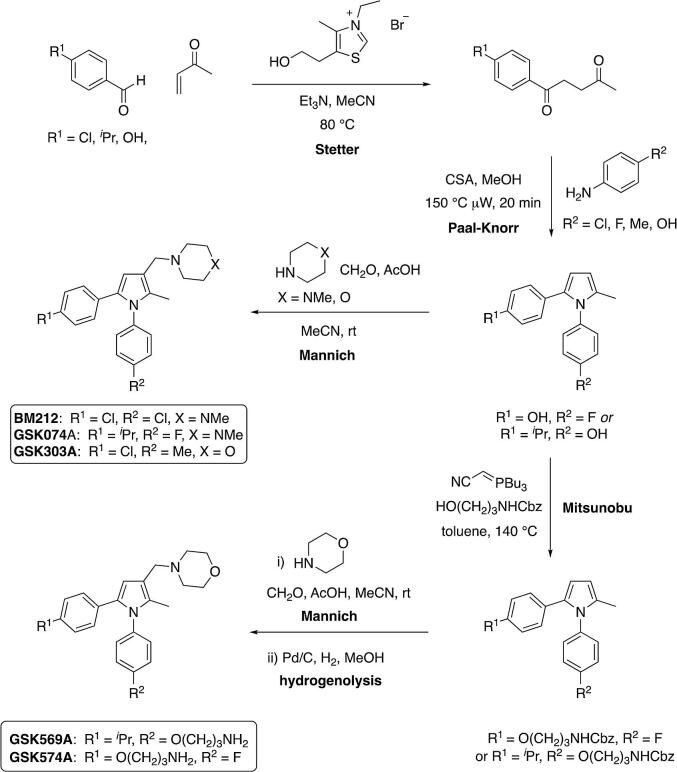
General synthetic route to BM212 and analogues used in this study.

The pyrrole compounds used in this study are collected in Fig. 1 and were synthesised according to General Scheme 1. In all cases, a Stetter reaction was used to provide a 1,4-diketone, which was employed in a Paal-Knorr synthesis to provide the corresponding pyrrole. A Mannich reaction was used to introduce the heterocycle at C3, either a piperazine in the case of BM212 and GSK074A, or a morpholine in the case of GSK303A, GSK569A and GSK574A. The 3-aminopropyl linker in GSK569A and GSK574A was introduced as its Cbz carbamate by a Mitsunobu reaction prior to the Mannich step and the primary amine then released by hydrogenolysis.

Whilst BM212 was the most potent first-generation inhibitor reported by Deidda et al. (1998), subsequent structure–activity studies led to the development of more potent analogues with improved physicochemical properties and drug-like parameters (Biava et al., 1999, Biava et al., 2004, Biava et al., 2005, Biava et al., 2006a, Biava et al., 2006b, Biava et al., 2008, Biava et al., 2010, Poce et al., 2013, Venditti et al., 2017, Poce et al., 2018). For example, analogues in which the *N-*methylpiperazine unit in BM212 was replaced with a thiomorpholine generally displayed improved potency and reduced cytotoxicity (Biava et al., 2004, 2005), while the corresponding morpholine analogues, such as GSK303A, displayed even better drug-like properties, notably improved *in vitro* microsomal stability, and physicochemical properties (lower lipophilicity) (Poce et al., 2013). Exchanging the 4-chloro substituents in the phenyl rings at N1 and C5 of BM212 with other substituents also provided a means to further increase potency, and critically, afforded potent inhibitors, for example, the thiomorpholine (Biava et al., 2008) and morpholine (Poce et al., 2013) analogues of GSK074A, with improved safety profiles. In line with the activity reported by Bavia and co-workers for these (thio)morpholine analogues, GSK074A, an *N*-methylpiperazine analogue, also proved to be a potent inhibitor and more potent than BM212 (Table 2).

**Table 2:** *M. bovis* BCG MIC values of BM212 and analogues used in this study. *MIC90 and MIC99 values determined at GSK DDW at Tres Cantos, Madrid, Spain. ND is not determined.

Compound name	MIC90 (μg/mL)*	MIC99 (μg/mL)*
BM212	1	ND
GSK074A	0.37	ND
GSK303A	0.16	ND
GSK569A	ND	20.7
GSK574A	ND	1.45

Attaching an inhibitor to beads for chemoproteomics experiments has the potential to affect target engagement. To mitigate such issues, we introduced a functionalised linker for bead attachment into two positions of the BM212 analogues. Using the morpholine analogue of GSK074A (Poce et al., 2013), a 3-aminopropyl linker was attached through the 4-position of the phenyl substituent at N1 to provide GSK569A (F to O(CH_2_)_3_NH_2_ substitution) and through the 4-position of the phenyl substituent at C5 to provide GSK574A (*^i^*Pr to O(CH_2_)_3_NH_2_ substitution).

These two primary amine-functionalised BM212 analogues were used in chemoproteomic profiling to elucidate further the mode of action of these potential MmpL3 inhibitors (Table 2, and Supplementary Tables 1 and 2). To this end, GSK569A and GSK574A were immobilised on Sepharose beads to generate bead matrices. A quantitative competition-based approach was applied to distinguish between proteins binding to the immobilised compound and background. The test compound, GSK074A, was spiked into aliquots of protein extract (*M. bovis* BCG extract) over a range of concentrations (40 µM, 1:3 dilutions) and competed with the immobilised analogues for binding to the target proteins. Matrix-bound proteins were eluted, trypsinised and subsequently encoded with isobaric mass tags (TMT10), enabling relative quantification by LC-MS/MS. Dose-dependent reduction from bead binding allowed the determination of concentrations of half-maximal binding (IC_50_). Apparent dissociation constants (*K*_d_^app^) were derived from the IC_50_ values by taking into account the amount of target sequestered by the affinity-matrix using the Cheng-Prusoff relationship (IC_50_ / *K*_d_^app^ correction factor) as determined in sequential binding experiments (Bantscheff et al., 2011). Duplicate vehicle controls, sequential binding experiments and a 6-point dose–response (40 µM, 1:3 dilutions) were analysed in a single 10-plexed mass spectrometric experiment (Eberl et al., 2019).

**Fig. 2:**
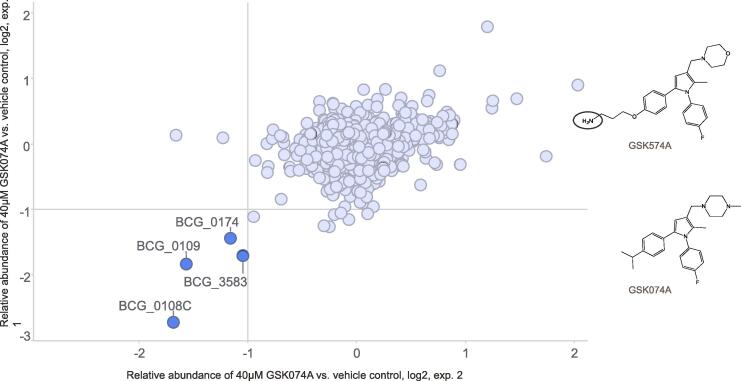
Chemoproteomic profiling of GSK074A identified four proteins as potential targets. A structural analogue of GSK074A, GSK574A, was attached to NHS-Sepharose beads. The bead matrix was incubated with *M. bovis* BCG protein extract in the absence or presence of 40 µM GSK074A. Targets were identified by substantially reduced binding to the bead matrix in the presence of GSK074A compared to vehicle control (>2-fold change, indicated by lines in the graphs). Chemical structures of utilised compounds are shown (region for attachment to Sepharose beads is indicated by a circle).

**Fig. 3:**
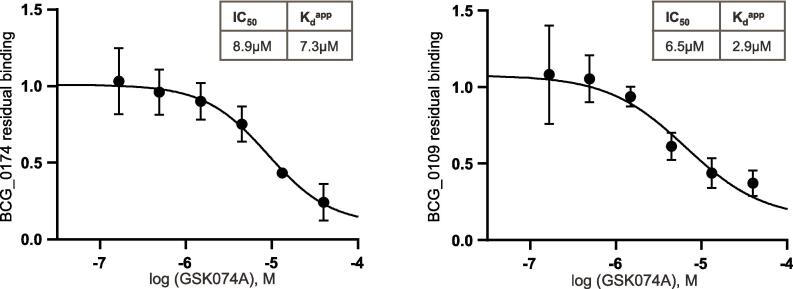
IC_50_ and apparent *K*_d_ values of GSK074A for BCG_0174 and BCG_0109. Bead matrices were generated by immobilising two amine-functionalised analogues, GSK569A and GSK574A, to NHS-Sepharose beads. The bead matrices were incubated with aliquots of *M. bovis* BCG protein extract containing different concentrations of GSK074A (starting at 40 µM, 1:3 dilutions). IC_50_ values were determined by quantifying the reduction of binding to the bead matrices by quantitative mass spectrometry and subsequent curve fitting. Apparent *K*_d_ values (Supplementary Tables 1 and 2) were calculated from the generated IC_50_ values (described in Results). IC_50_ values for BCG_0174 (uncharacterised protein) and BCG_0109 (EthR2) competition were averaged from 6 independent experiments and standard deviations were calculated (BCG_0174: IC_50_ 8.9 µM +/–1.1 µM; BCG_0109: IC_50_ 6.5 µM +/–1.5 µM).

GSK074A was analysed on both bead matrices and inhibited four proteins from bead binding (in at least 2 experiments with the same bead-matrix): BCG_3583, BCG_0174, BCG_0109 and BCG_0108c (Fig. 2). In total, 10 experiments were performed with the four bead matrices (Supplementary Tables 1 and 2). Dose-dependent competition for BCG_0109 (EthR2) was observed in 6 experiments. Competition for BCG_0174, enabling calculation of IC_50_ values, was observed in 6 out of 10 experiments (competition on both bead matrices). The generated apparent *K*_d_ values for the potential target proteins, BCG_0174 and BCG_0109, were 7.3 µM and 2.9 µM, respectively. These values are high (Fig. 3), compared to the activity of the test compound GSK074 against *Mtb* with a MIC_90_ value of 0.9 µM. Competition for BCG_3583 and BCG_0108c was less consistently observed (3 out of 10 experiments, Supplementary Tables 1 and 2) and did not allow determination of IC_50_ values in replicate experiments.

BCG_3583 (acetoacetate decarboxylase domain-containing ortholog to Rv3519) and BCG_0174 (SnoaL-like domain-containing ortholog to Rv0138) are both uncharacterised proteins. BCG_0109 and BCG_0108c are the orthologues to Rv0078 and Rv0077c, which are also termed EthR2 and EthA2, respectively (Blondiaux et al., 2017). Rv0078 regulates the expression of Rv0077c by binding within its intergenic region (Blondiaux et al., 2017), in analogy to EthR and EthA (Rv3855 and Rv3854c). EthA is regulated by the transcriptional repressor EthR (Engohang-Ndong et al., 2004). Inhibitors of EthR stimulate the transcription of the *ethA* gene, which improves the bioactivation of the ethionamide (ETA) (Flipo et al., 2011). Interestingly, deletion of the *ethA*-*ethR* locus did not impair the general fitness of mycobacteria (Ang et al., 2014), but there are no data available on the inhibition of both EthR2 and EthA2. In analogy to EthR and EthA, and also Rv3519/Rv0138 (non-essential proteins by TraSH) it seems unlikely that the inhibition of these proteins alone leads to the anti-bactericidal activity of the BM212 compound series. However, binding may reflect the off-target nature of the BM212 compound series with the possibility that BM212 may synergise with ETA via EthR/EthA, which was further explored.

### Biochemical studies

**Fig. 4:**
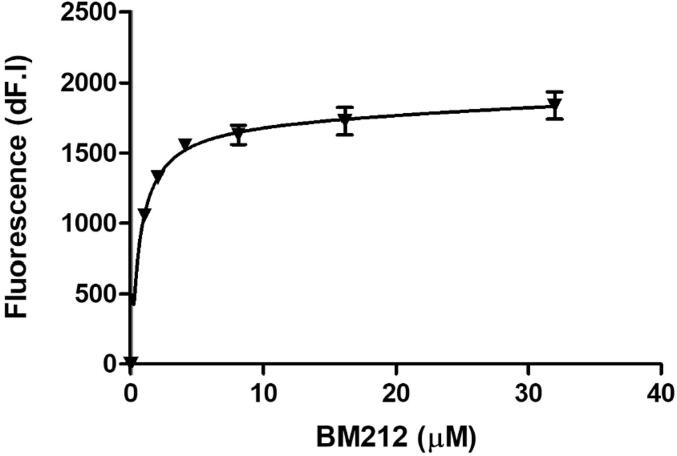
Fluorescence quenching of EthR2 tryptophan residues with increasing concentrations of BM212, *K*_D_ = 0.66 µM.

The repressor protein, EthR2, was overexpressed with a histidine tag in *E. coli* DE3 cells from a pET vector, and purified via a nickel column. The purified EthR2 repressor protein was subjected to tryptophan fluorescence binding assays to determine BM212 binding. Addition of BM212 to the EthR2 protein quenched tryptophan emission fluorescence at 355 nm, such that a saturation ligand-binding curve could be generated and the *K*_D_ calculated as 0.66 µM (Fig. 4).

### Microbiological studies

**Fig. 5:**
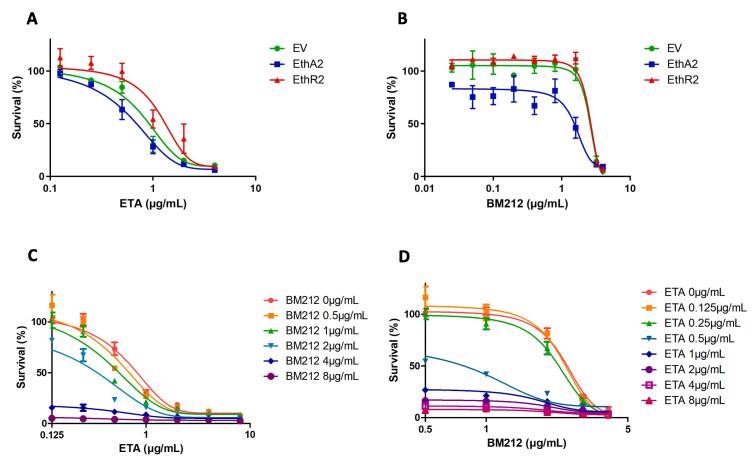
MICs of *M. bovis* BCG treated with ETA and BM212. A and B., ETA and BM212 survival curves, respectively for *M. bovis* BCG expressing an empty vector plasmid (EV), EthA2 and EthR2, showing lower tolerance of both ETA and BM212 by EthA2 and a modest increased tolerance of ETA by EthR2. Plotted as the mean of triplicate data with error bars showing SEM. C and D., Survival curves of ETA and BM212 for *M. bovis* BCG expressing an empty vector plasmid, showing the dose response when both drugs are applied in combination at different concentrations. Plotted as the mean of triplicate data with error bars showing SEM.

MIC assays were carried out to determine the MIC_90_ of ETA and BM212 in liquid media, and to establish whether any synergistic or ETA boosting interactions occurred when the inhibitors were applied in combination at sub-lethal concentrations. MICs were determined for wild type (WT) *M. bovis* BCG and for *M. bovis* overexpressing EthA2 and EthR2 (Rv0077c and Rv0078, respectively). Inhibition by both ETA and BM212 was demonstrated, both with MIC_90_ values against WT BCG of 4 µg/mL (Fig. 5A and B, conducted at the University of Birmingham).

Quantitative determination of the interaction between ETA and BM212 was achieved using the fractional inhibitory concentration (FIC) index. FIC values, based on the concentrations needed to inhibit 75% of growth of ETA and BM212, which were 2 µg/mL and 3 µg/mL, respectively, were calculated using equation 1 to determine whether the inhibitors act synergistically (∑FIC < 0.5), additively/indifferently (∑FIC = 0.5 – 4) or antagonistically (∑FIC > 4). For WT *M. bovis* BCG the FIC value was 0.83, indicating an additive/indifferent effect.

## Discussion

Chemoproteomic profiling of the BM212 series analogue, GSK074, using immobilised Sepharose beads, identified BCG_0108c, BCG_0109, BCG_0174 and BCG_3853 as potential targets. These corresponded to rv0077c (*ethA2*), *rv0078* (*ethR2*), *rv3519* and *rv0138* in *Mtb*, the latter two being uncharacterised proteins. Recently, Rv0077c and Rv0078 (designated EthA2 and EthR2) were discovered to be functional analogues of EthA and EthR (Baulard et al., 2000, DeBarber et al., 2000, Blondiaux et al., 2017), two proteins involved in the activation of the second-line antitubercular drug ETA, which is a prodrug and a structural analogue of isoniazid (INH). In its activated form, ETA and nicotinamide adenine dinucleotide (NAD+) are covalently bound and together inhibit InhA, an enoyl-[acyl-carrier-protein] (Enoyl-ACP) reductase, which is also targeted by isoniazid (Banerjee et al., 1994). InhA is involved in mycolic acid biosynthesis, forming part of the type II fatty acid synthase system (FAS-II), and plays an essential role in the extension of short-chain fatty acids (Marrakchi et al., 2000, Batt et al., 2020).

Despite being structural analogues, the occurrence of cross-resistance to both ETA and INH is relatively low due to their different activation pathways (Zhang et al., 1992, Baulard et al., 2000). The activation of ETA is catalysed by the monooxygenases EthA and EthA2, the expression of which is regulated by the transcriptional repressors, EthR and EthR2, respectively (DeBarber et al., 2000, Vannelli et al., 2002). The effects of EthR and EthA on ethionamide efficacy has been extensively researched. The genes responsible for encoding these proteins, *ethA* and *ethR* (*rv3854c* and *rv3855*)*,* share a promotor region which is repressed by EthR (Baulard et al., 2000, Engohang-Ndong et al., 2004). Inhibition of EthR improves ETA potency by increasing the rate of ETA activation by EthA and several small molecules have been discovered to successfully boost EthA2 expression by inhibiting EthR2 repression of the operon (Blondiaux et al., 2017).

There have recently been promising developments in the application of pharmacokinetic enhancers or ‘drug boosters’, which are intended to increase the efficiency or bioavailability of a drug and therefore reduce the required dose without necessarily directly targeting the causative agent of a disease (Krauß and Bracher, 2018). Amongst, the most well documented boosters are the antiretroviral enhancers, Cobicistat and Ritonavir (Larson et al., 2014), which have been used to tackle poor oral bioavailability of HIV therapies. Both drugs act as cytochrome P450 inhibitors, reducing the metabolism of antiretroviral drugs (Danner et al., 1995, Hsu et al., 1998). Other pharmacokinetic enhancers include β-lactamase inhibitors such as the PBP2 binding β-lactam enhancer zidebactam, which, in combination with the β-lactam cefepime, is active against MDR Gram-negative bacteria (Bhagwat et al., 2019, Bhagwat et al., 2021, Moya et al., 2020). β-Lactam enhancers have also been applied to drug-resistant strains of *Mtb,* where susceptibility to the β-lactam carbapenem has been successfully restored by combinatorial treatment with the β-lactamase inhibitor clavulanic acid (Kurz and Bonomo, 2012, Ramón-García et al., 2016). Existing boosters for ETA are primarily synthetic compounds, which have been developed as a result of fragment-based screening for small-molecule ligands that inhibit EthR. Little work however has been carried out to screen existing drugs which might be repurposed as combination therapies with ETA.

The identification of EthA2 and EthR2 has revealed a new pathway for ETA activation, and with it new opportunities to apply boosting compounds. *In vitro* SMARt (Small Molecule Aborting Resistance) screening revealed that SMARt-420 interacts directly with EthR2 and triggers the overexpression of EthA2. When used in combination with ETA, SMARt-420 restored sensitivity to ETA in a strain with a mutated EthA (Blondiaux et al., 2017).

**Fig. 6:**
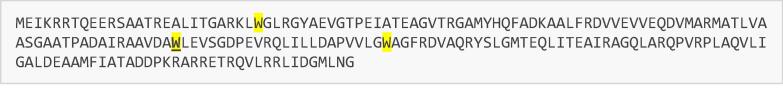
Amino acid sequence of EthR2 (Rv0078) with tryptophan residues (W) highlighted in yellow. The tryptophan residue near to the binding site of several known EthR2 inhibitors is in bold and underlined (Prevet et al., 2019). (For interpretation of the references to colour in this figure legend, the reader is referred to the web version of this article.)

Using the intrinsic tryptophan fluorescence of EthR2, we were able to demonstrate fluorescence quenching in the presence of BM212, supporting the chemoproteomic profiling results identifying EthR2 as a target and ligand for BM212. The EthR2 protein has three tryptophan residues (Fig. 6), one of which is near to the binding site of several known inhibitors of EthR2 (Prevet et al., 2019). However, there is insufficient evidence from the intrinsic tryptophan fluorescence binding assays to predict whether BM212 also binds to this site as it is unclear which tryptophan residue(s) is affected by ligand binding. The bead immobilisation assays indicated that EthR2 bound consistently to GSK074A (of the BM212 series) in multiple experiments with a *K*_D_ of 2.9 µM, while the *K*_D_ of BM212 calculated from the EthR2 tryptophan fluorescence assay was 0.66 µM. We investigated the potential for BM212 to inhibit growth in *M. bovis* BCG both alone and in combination with ETA, to identify if there is a synergistic relationship between the two compounds. When BM212 and ETA were applied in combination to *M. bovis* BCG there was no identifiable synergy. The FIC method for quantitative determination of drug interactions, indicated an additive or indifferent effect between the two drugs for the BCG pMV261 empty vector control, which is comparable to a WT strain.

Exploration of pharmacokinetic enhancers in the treatment of TB is an important step in the effort to reduce treatment durations and to combat drug resistance. The prospect of losing more drugs from the anti-TB armoury to resistant strains of TB is alarming and a multifaceted approach is needed to avoid losing the efficacy of the current poly-therapeutic regimens that we rely on. The discovery of new drug boosters can occur through fragment-based screening approaches, as demonstrated by the work on SMARt-420, but there is also scope to investigate interactions between existing drug candidates, such as BM212 and ETA. Here we have demonstrated the value of revisiting drugs with ambiguous targets, such as MmpL3, in an attempt to find alternative targets and the study of off-target effects.

### CRediT authorship contribution statement

**Alice R. Moorey:** Conceptualization, Writing – original draft, Writing – review & editing, Visualization. **Alejandro Cabanillas:** Conceptualization, Writing – original draft, Writing – review & editing, Visualization. **Sarah M. Batt:** Conceptualization, Writing – original draft, Writing – review & editing, Visualization. **Sonja Ghidelli-Disse:** Conceptualization, Writing – original draft, Writing – review & editing, Visualization. **Beatriz Urones:** Conceptualization, Data curation, Writing – review & editing. **Olalla Sanz:** Conceptualization, Data curation, Writing – review & editing. **Joel Lelievre:** Conceptualization, Writing – original draft, Writing – review & editing, Visualization. **Marcus Bantscheff:** Conceptualization, Writing – original draft. **Liam R. Cox:** Conceptualization, Writing – original draft, Writing – review & editing. **Gurdyal S. Besra:** Conceptualization, Writing – original draft, Writing – review & editing.

## Declaration of Competing Interest

The authors declare that they have no known competing financial interests or personal relationships that could have appeared to influence the work reported in this paper.
